# Impact of Surface Changes and Microbial Adhesion on Mucosal Surface Finishing of Resin Denture Bases by Shot Blast Polishing Using Viscoelastic Media

**DOI:** 10.3390/ma15062275

**Published:** 2022-03-19

**Authors:** Yusuke Yamashita, Yasuhiro Nishi, Mamoru Murakami, Kae Harada, Masahiro Nishimura

**Affiliations:** 1 Department of Oral and Maxillofacial Prosthodontics, Field of Oral and Maxillofacial Rehabilitation, Kagoshima University Graduate School of Medical and Dental Sciences, Kagoshima 890-8544, Japan; k2679941@kadai.jp (Y.Y.); hkae@dent.kagoshima-u.ac.jp (K.H.); mnishi@dent.kagoshima-u.ac.jp (M.N.); 2 Department of Removable Prosthodontics and Implant Dentistry, Advanced Dentistry Center, Kagoshima University Hospital, Kagoshima 890-8544, Japan; kaku@dent.kagoshima-u.ac.jp

**Keywords:** denture polishing, surface roughness, denture surface, microbial adhesion

## Abstract

Surface changes and microbiological effects following shot blast polishing with viscoelastic media of the mucosal surface of resin denture bases were examined. Average surface roughness (Ra) and the depth of surface removal of specimens were measured over time, and the clinical number of microbial adhesions on the mucosal surface of dentures was clinically assessed. The results obtained showed no changes in Ra after 20 s of polishing, Ra of <0.2 µm, and a depth of surface removal < 20 µm. This method of finishing did not affect the fit of the mucosal surface of the dentures. Furthermore, the adhesion of microorganisms to the mucosal surface of dentures was significantly suppressed. Shot blast polishing with viscoelastic media is useful for finishing the mucosal surface of resin denture bases.

## 1. Introduction

Removable dentures have been fabricated from PMMA resin using a fluid resin and the mold-filling method for 80 years, and PMMA resin is still the main material used due to its ease of manufacture, repair, and polishing as well as good physicochemical properties and acceptable esthetics [1,2]. Since PMMA resin has low flexural strength and low flexibility despite its many advantages [3,4], high-strength polysulfone resin and flexible polyamide resin have been developed [5,6]. However, any material for use as an intraoral prosthetic device requires a smooth surface from the viewpoint of microbial plaque retention, staining, oral health, and patient comfort [7,8,9]. To achieve ideal aesthetics and oral hygiene, the surface of a dental prosthesis needs to be as smooth as possible, even if the patient manages the dental prosthesis properly. Surface roughness (Ra) plays a key role in plaque accumulation and bacterial adhesion to denture base materials. In vitro experiments revealed larger amounts of *Candida albicans* on roughened surfaces than on smooth surfaces [10,11]. A recent study reported that bacteria and yeast cells both adhered to surfaces of increasing roughness, and also that the strength of microbial attachment was more important than the amount [12]. The roughness of denture base materials is affected by material properties and polishing techniques [1,13,14]. Mechanical polishing achieves a smoother polished surface than chemical polishing [15], while conventional laboratory polishing provides a smoother polished surface than chairside polishing [1,16]. The polished surface of the denture base, which is not in contact with the mucosa of the ridge, is sufficiently polished by conventional methods, such as that involving polishing wheels. On the other hand, although the mucosal surface of the denture base is important for compatibility, sufficient polishing is not often performed. The mucosal surface of the denture base is always in contact with the mucous membrane of the edentulous alveolar ridge and transmits a functional load while the denture is in place; however, this is not a sanitary area due to the lack of saliva. A consensus has not yet been reached on polishing methods for the mucosal surface of the denture base. However, from the viewpoint of suppressing the adhesion of microorganisms, Ra of the mucosal surface of the denture base needs to be as low as possible.

Polishing using the recently developed shot blasting method, which shoots viscoelastic composite polishing media, enables mirror polishing without inducing morphological changes in the object being polished, such as metal [17]. The application of this polishing method to the mucosal surface of denture base resin in order to obtain a smooth surface with negligible morphological changes may successfully suppress the adhesion of denture plaques.

Therefore, the present study investigated changes in the physical properties of the mucosal surface of denture base resin in vitro and the microbiological effects of polishing in vivo when the shot blasting method using viscoelastic composite polishing media was performed.

## 2. Materials and Methods

### 2.1. Polishing by the Shot Blasting Method 

**Figure 1:**
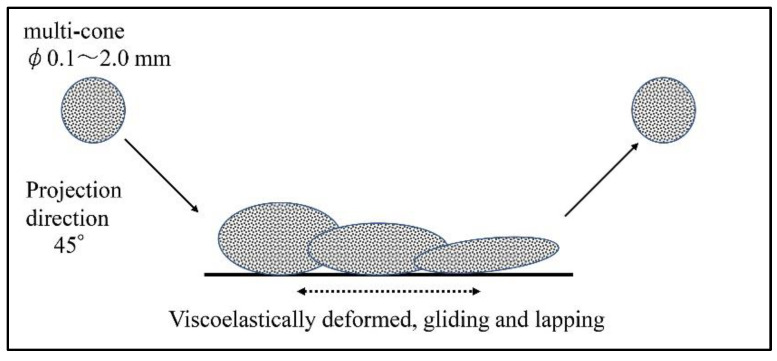
SBVM polishing image; polishing by shot blasting composite polishing media (multi-cone, Yamashita Works, Kyoto, Japan) consisting of viscoelastic food proteins containing water and diamond particles as abrasive grains.

The effects of a novel polishing method for denture mucosal surfaces on the physical properties of the surface of denture base resin as well as its clinical microbiological effects were examined in the present study. This polishing method (AERO LAP^®^ https://www.yamashitaworks.co.jp/product/aerolap/ accessed on 17 March 2022) is performed using a shot blast polishing machine that shoots a viscoelastic composite polishing medium made of food proteins containing diamond particles. Figure 1 shows the mechanism of this polishing method. Shot-blast viscoelastic media (SBVM) polishes while deforming on the surface of the object to be polished. A shot blast polishing device (T-100, Yamashita Works, Kyoto, Japan) and polishing media containing #20,000 diamond particles were used in the present study, and this polishing method is referred to as “SBVM polishing” hereafter. In the present study, the shot blasting angle relative to the objects and distance to objects in SBVM polishing was generally set to 45° and 5 cm, respectively.

### 2.2. Sample Preparation

Heat-cured polymethylmethacrylate denture base resin (ProBase Hot, Ivoclar Vivadent AG, Schaan, Liechtenstein) was used in the present study. The material was manipulated and polymerized according to the manufacturer’s instructions. Specimens were cured using a stainless-steel mold with dimensions of 10 × 30 × 1.5 mm and a Teflon plate. Thirty specimens were prepared and the surfaces of 10 specimens were sanded sequentially with 400-grit, 800-grit, and 1500-grit abrasive papers (400-grit, 800-grit, and 1500-grit specimens, respectively). The size of each specimen was confirmed using digital calipers and specimens sanded with each abrasive paper were ground and polished to ensure uniform roughness. Specimens were stored in water at 37 °C for 24 h to release residual monomers.

### 2.3. Evaluation 1 of Physical Properties: Ra

In three specimens each for the three polishing conditions (400-grit, 800-grit, and 1500-grit), changes in Ra by polishing were measured every 10 s for 50 s. The Ra of specimens was measured using a surface profilometer (Surfcom 130A, Accretech, Tokyo, Japan), which scanned a sample length of 6.0 mm at 0.6 mm/s with a cut-off value of 0.8 mm. Three scans were recorded at three different locations for each specimen, and the average of three mean Ra measurements was selected as the score for each specimen. Microscopic observations were conducted on 400-grit and 1500-grit specimens before and after polishing for 50 s using a scanning electron microscope (SEM, JSM-5510LV, JEOL, Tokyo, Japan) operating at an accelerating voltage of 20 kV.

### 2.4. Evaluation 2 of Physical Properties: Depth of Surface Removal

Reductions due to polishing, namely, the depth of surface removal due to polishing, were measured. In three specimens each for the three polishing conditions (400-grit, 800-grit, and 1500-grit), the depth of surface removal due to polishing every 10 s was measured from before polishing to 50 s. The depth of surface removal of specimens was assessed by measuring the distance to the surface of the specimens using a CCD laser displacement sensor (LK-G35, Keyence Co., Osaka, Japan) before and after polishing. This measurement was performed at the center point of the specimen and two points at a position 3 mm in the long axis direction from the center point using the measuring apparatus with the XY table, for a total of three points, and the average of these values was used as the measured value. A jig to return specimens to the fixed position of the measuring apparatus before and after polishing was manufactured. Distance measurements were accomplished by attaching and detaching this jig at the fixed position on the XY table of the apparatus.

### 2.5. Microbiological Evaluation

Twenty-one edentulous individuals (13 males, 8 females; mean age: 74.3 years; S.D.: 6.8 years; range: 57–85 years) agreed to participate in the present study. All participants were patients who were able to use the new complete upper and/or lower dentures made of acrylic resin satisfactorily after they had been fabricated and adjusted at the Removable Prosthodontics and Implant Dentistry, Advanced Dentistry Center, Kagoshima University Hospital. The aim of the present study was explained to the participants using a document approved by the Clinical Study Ethics Committee of Kagoshima University Hospital (#190226-Epidemiology), and written consent was obtained. The dentures examined included 22 upper and 11 lower dentures (33 dentures in total), which no longer needed to be adjusted at the time of visits. They were rinsed in tap water, immersed in a 1.5% sodium hypochlorite solution (Improsterin+, Taihei Chemical Co., Osaka, Japan) for 5 min, and the mucosal surface of the right lateral half of the examined denture was then polished using the SBVM polishing method for approximately 3 min as the adequate time to obtain the mirror polishing. After polishing, the denture was returned to the patient for normal use, and microbiological examinations were performed at a visit two weeks later. Participants were instructed to clean their dentures daily with a denture cleanser.

To collect denture plaque, dentures were removed, lightly rinsed with running water to remove any saliva, and then air dried. Denture plaques were collected by a single dental examiner who swabbed the right side (polished side) and left side (non-polished side) of the denture mucosal surface using sterile swabs that had been immersed in phosphate-buffered saline (PBS) (Fukifuki Check II^®^, Eiken Chemical, Tochigi, Japan). Based on previous findings showing that the distribution of plaque on the mucosal surface of complete dentures varied between different denture regions, but was bilaterally symmetric in different complete dentures [18], the left and right halves of the denture were swabbed twice in their entirety. Each sterile swab was vortexed in 10 mL PBS in a plastic bottle and the resultant samples were transported to a laboratory, at which they were plated and incubated within 5 h of sampling and used to identify microorganisms. The microorganisms to be cultured were Gram-positive cocci, Gram-negative bacilli, and *Candida* spp., which are considered to be the causative agents of aspiration pneumonia. Specimens were inoculated onto sheep’s blood agar plates (Try/Soy Blood Agar (Sheep) No.2; Kyokuto Pharmaceutical Industrial, Tokyo, Japan) and CHROM agar Candida plates (CHROMagar Candida; Kanto Chemical, Tokyo, Japan). These plates were incubated at 37 °C for 48 h under aerobic conditions. Microorganisms were presumptively identified based on the colors and morphological features of colonies, and the number of each type of colony was counted. The number of all colony-forming units per mL was assessed on the polished and non-polished sides of each experimental denture. 

### 2.6. Analysis 

A two-way repeated measures analysis of variance (ANOVA) and multiple comparison test were used to compare differences in Ra and the depth of surface removal (n = 5). 

A comparison of microbial counts between the polished and non-polished sides was performed with the Wilcoxon signed-rank test and the Mann–Whitney U test. All statistical analyses were performed using a statistical analysis application (SPSS Statistics ver.26, IBM Japan Ltd., Tokyo, Japan). *p* values ≤ 0.01 were considered to be significant.

## 3. Results

### 3.1. Physical Properties

**Figure 2:**
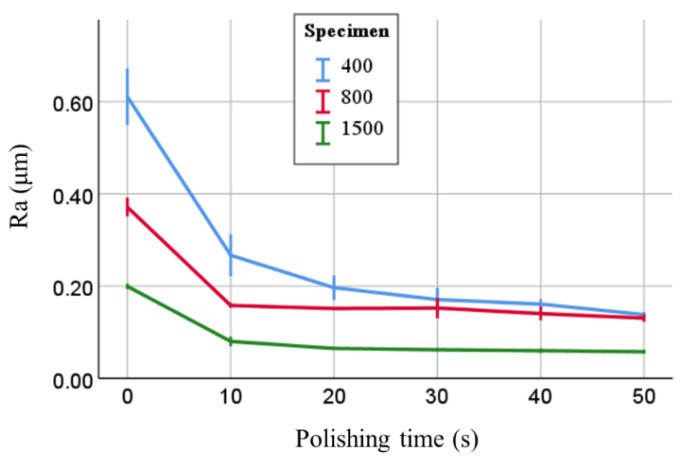
Changes in mean surface roughness with polishing times and specimen types. Error bar indicates ±SD.

**Figure 3:**
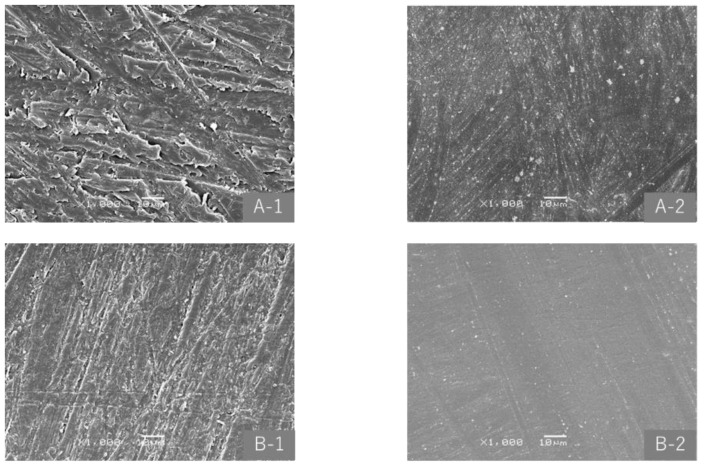
SEM images (×1000) before and after SBVM polishing. (**A-1**) 400-grit specimen before polishing; (**A-2**) 400-grit specimen after polishing; (**B-1**) 1500-grit specimen before polishing; and (**B-2**) 1500-grit specimen after polishing.

As shown in Figure 2, the Ra of 400-grit, 800-grit, and 1500-grit specimens markedly decreased after polishing for 20 s, and showed almost no change thereafter. Differences in Ra among the three types of specimens became smaller as the polishing time became longer; however, Ra after polishing appeared to be affected by that before polishing. In the two-way repeated measures ANOVA, with the factors specimen type and polishing time, both factors significantly affected Ra (both *p* < 0.001), and there was an interaction between the two factors (*p* < 0.001). In the multiple comparison test, significant differences were observed between any two of the three specimens (each *p* < 0.001), but not after a polishing time of 20 s or longer. Figure 3 shows SEM images of 400-grit and 1500-grit specimens before and after polishing. Surface smoothing by polishing was observed in specimens. 

**Figure 4:**
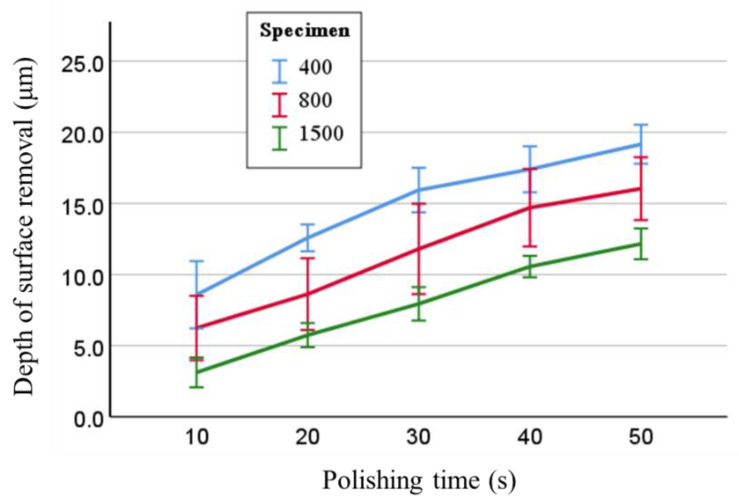
Mean depth of surface removal by polishing times and specimen types. Error bar indicates ±SD.

The depth of surface removal increased in proportion to the polishing time, and the average depth of surface removal in 20 s when the change in Ra became small were 12.6, 8.6, and 5.7 μm for 400-grit, 800-grit, and 1500-grit specimens, respectively (Figure 4). In the two-way repeated measures ANOVA, with the factors specimen type and polishing time, both factors significantly affected the depth of surface removal (both *p* < 0.001), and no interaction was observed between the two factors. In the multiple comparison test, significant differences were noted in the depth of surface removal between any two of the three specimens (*p* < 0.001 each) and in each specimen at all polishing times.

### 3.2. Microbiological Evaluation

**Figure 5:**
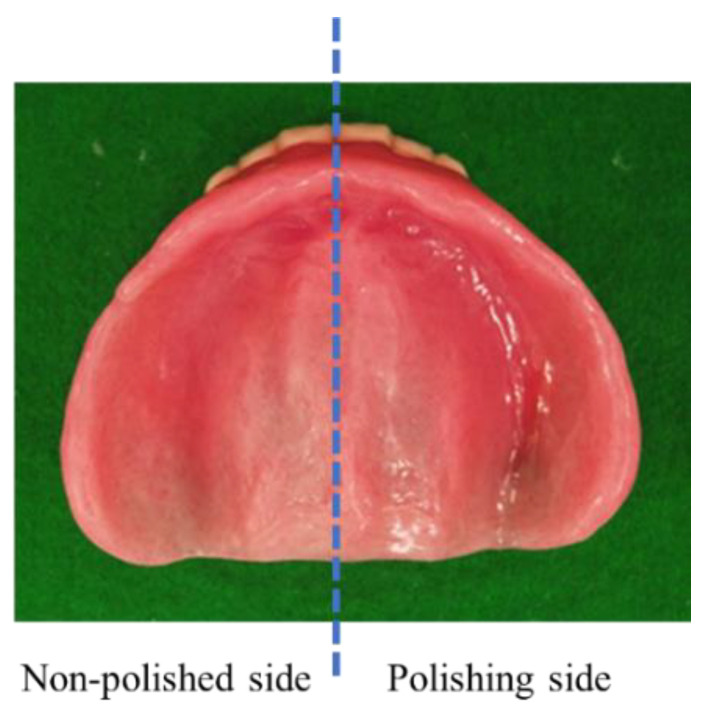
The mucosal surface of a new denture with the right half polished and the left half not polished.

**Figure 6:**
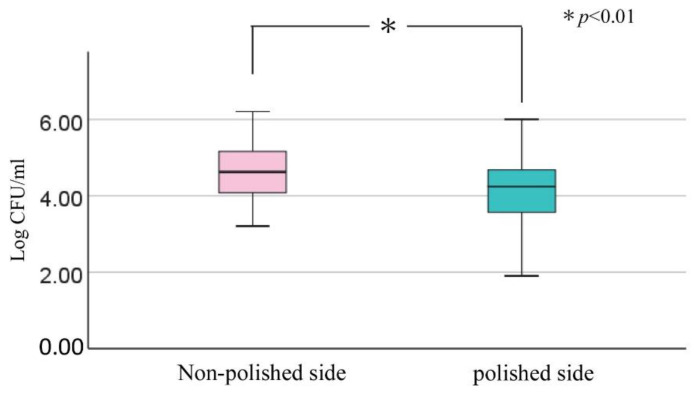
Comparison of total microbial adhesion between half sides of the denture mucosal surface with and without polishing. In each box plot, the central point represents the median, the rectangle gives the interval between the 25% and 75% percentiles. The whiskers represent the lowest and highest value in the 25% percentile minus 1.5IQR and 75% percentile plus 1.5IQR regions, respectively.

Figure 5 shows a denture with SBVM polishing on the right half of the mucosal surface. After denture adjustment, the right half was subjected to SBVM polishing while the left half was not. Light reflected on the resin surface on the right half and it was in a mirror-polished state. A comparison of the number of microbial adhesions with and without SBVM polishing was performed on 32 dentures, including the upper and lower jaws, from 21 patients. There was no significant difference in microbial adhesion between the upper and lower dentures in each agar plate and all microorganisms. The number of microbial adhesions was significantly smaller on the polished side than on the non-polished side (Figure 6).

## 4. Discussion

In the present study, the Ra of the denture base resin was reduced by polishing with SBVM but hardly changed after polishing for about 20 s. Furthermore, the depth of surface removal was the greatest for 400-grit specimens with the largest Ra, at approximately 12 μm; however, this was not considered to be a large depth of surface removal, because it is smaller than 30 μm, which is the minimum gap between abutment teeth and even highly fitting cast crowns [19]. Therefore, SBVM polishing on the mucosal surface of resin dentures does not have a negative impact on compatibility. Based on the present results, a larger Ra on the mucosal surface of denture bases is associated with a greater depth of surface removal; however, as described above, the depth of surface removal did not affect the suitability of the denture base. Limited information is currently available on the effects of polishing of the mucosal surface of denture bases; however, changes in the shape of the mucosal surface of denture bases due to barrel finishing were reported in the 1990s in Japan [20,21]. Barrel finishing has also been used for cobalt–chromium alloy casting [22], but is not popular nowadays because the whole denture is polished at the same time and it is difficult to polish a specific part. However, SBVM polishing has the advantage of targeted polishing in a limited area without the scattering of dust.

It is a limitation of this study that the surface roughness of the denture and the amount of removal of the denture surface could not be measured in vivo. However, since the denture was polished for as short a time as possible (about 3 min) while obtaining a smooth surface on the half side with the denture mucosal surface (about 7 to 8 times the area of the in vitro specimen), the amount of denture surface removed was considered not to affect the fit of the denture as described above.

In the present study, a significant difference was observed in the number of adhering microorganisms between the polished and non-polished sides of the mucosal surface of complete resin dentures, demonstrating the effectiveness of SBVM polishing. This result is consistent with previous findings showing less microbial adhesion with lower Ra [12,14,23,24]. However, the number of microorganisms that attached to the polished side did not markedly decrease. Based on in vivo studies [9,25], the threshold of Ra for microbial adhesion in clinically acceptable prostheses containing denture base resin is 0.20 µm. The Ra of 400-grit, 800-grit and 1500-grit specimens after SBVM polishing in the present study was 0.2 µm or less; however, Ra is considered to be larger in clinical dentures. Therefore, we attempted to measure the Ra of the mucosal surface of some new dentures and the plaster surface of working models that could be measured, although one study reported general Ra in vitro [26]. The results obtained showed that the Ra of the unpolished mucosal surface of new dentures after polymerization ranged between 0.9 and 2.8 µm. The plaster surface of the working model corresponding to the mucosal surface of dentures had a similar Ra. Although further studies are warranted, the Ra of the mucosal surface of resin dentures is considered to be reflected in that of the working model. The present study revealed that the Ra of the mucosal surface of denture bases after SBVM finishing was affected by that before polishing. Therefore, to reduce the Ra of the mucosal surface of denture bases, a treatment method that improves the surface texture of the working model is required in the future. On the other hand, methods that suppress the adhesion of microorganisms by treating the surface of denture bases have been reported [27,28,29,30,31]. A method of adhering or mixing nano-silver particles and a method of using a 2-methacryloyloxyethyl phosphorylcholine polymer or titanium dioxide for a resin base have been examined and are being applied to clinical settings. By combining chemical methods that suppress microbial adhesion with physical methods, such as SBVM finishing, an optimal method for preparing dentures will be possible in the future. However, since it has been reported that increased surface roughness of denture acrylic resin enhances retention of hyphae and yeast cells [32], even if the surface roughness of the mucosal surface of the denture base increases due to deterioration and the risk of denture stomatitis increases, this SBVM polishing will be able to improve the surface roughness.

In recent years, advances in digital dentistry have led to an increasing number of studies examining and verifying removable dentures fabricated with CAD/CAM and 3D printing [33,34,35]. Although the optical scanning of soft tissues, such as the ridge mucosa, will continue to be difficult, further technological advances may allow the Ra of these digitally produced dentures to be reduced. As a denture base material, PMMA resin will be widely used in digitalization in the future. Since it is desirable for dentures to have a smaller Ra, SBVM finishing will continue to be effective.

## 5. Conclusions

SBVM polishing showed that finishing could be achieved without affecting the morphology of the mucosal surface of denture bases and also suppressed the adhesion of microorganisms. However, the limitation of this study was the inability to identify the surface roughness of the in vivo denture mucosal surface that affects the adhesion of microorganisms. In addition, since Ra after polishing may be affected by that before polishing, improvements in the surface texture of denture bases and the addition of a method that suppresses microbial adhesion need to be considered in the future.

## Data Availability

The data presented in this study are available on request from the corresponding author.
